# *HOTAIR* as a Prognostic Predictor for Diverse Human Cancers: A Meta- and Bioinformatics Analysis

**DOI:** 10.3390/cancers11060778

**Published:** 2019-06-05

**Authors:** Halil Ibrahim Toy, Didem Okmen, Panagiota I. Kontou, Alexandros G. Georgakilas, Athanasia Pavlopoulou

**Affiliations:** 1 Ιzmir International Biomedicine and Genome Institute, Dokuz Eylül University, Balcova 35340, Turkey; ibrahim.toy@msfr.ibg.edu.tr (H.I.T.); didem.okmen@msfr.ibg.edu.tr (D.O.); 2 Department of Computer Science and Biomedical Informatics, University of Thessaly, Lamia 35131, Greece; pankontou@gmail.com; 3 DNA Damage Laboratory, Department of Physics, School of Applied Mathematical and Physical Sciences, Zografou Campus, National Technical University of Athens (NTUA), 15780 Athens, Greece; alexg@mail.ntua.gr

**Keywords:** *HOTAIR*, prognostic biomarker, survival, meta-analysis, cancer

## Abstract

Several studies suggest that upregulated expression of the long non-coding RNA *HOX transcript antisense RNA* (*HOTAIR*) is a negative predictive biomarker for numerous cancers. Herein, we performed a meta-analysis to further investigate the prognostic value of *HOTAIR* expression in diverse human cancers. To this end, a systematic literature review was conducted in order to select scientific studies relevant to the association between *HOTAIR* expression and clinical outcomes, including overall survival (OS), recurrence-free survival (RFS)/disease-free survival (DFS), and progression-free survival (PFS)/metastasis-free survival (MFS) of cancer patients. Collectively, 53 eligible studies including a total of 4873 patients were enrolled in the current meta-analysis. Pooled hazard ratios (HRs) with their corresponding 95% confidence intervals (CIs) were calculated to assess the relationship between *HOTAIR* and cancer patients’ survival. Elevated *HOTAIR* expression was found to be significantly associated with OS, RFS/DFS and PFS/MFS in diverse types of cancers. These findings were also corroborated by the results of bioinformatics analysis on overall survival. Therefore, based on our findings, *HOTAIR* could serve as a potential biomarker for the prediction of cancer patient survival in many different types of human cancers.

## 1. Introduction

The long non-coding RNAs (lncRNAs) are non-protein-coding RNAs ≥ 200 bp in length, transcribed by RNA polymerase II. LncRNAs can be capped, polyadenylated and spliced, but they lack a functional open reading frame. It is estimated that approximately 27% (i.e., up to 60,000) of the annotated genes in the human genome encode lncRNAs, while the number of protein-coding genes ranges from 20,000 to 25,000 [1,2]. They are largely involved in a myriad of cellular functions, regulating gene expression at the transcriptional, post-transcriptional, and epigenetic level [1,3]. LncRNAs have emerged as critical components of cancer pathophysiology, being involved in one or more hallmarks of cancer, such as proliferation and metastasis [4,5]. They can act either as oncogenes or tumor suppressors, or indirectly through interaction with oncogenes and tumor suppressors, such as MYC proto-oncogene (MYC) and tumor protein p53 (TP53), respectively [4,5]. 

One of the most well-studied lncRNAs is *HOX transcript antisense RNA* (*HOTAIR*) which is located within the *HOMEOBOX C* (*HOXC*) gene cluster on chromosome 12q13.13 [6]. *HOTAIR* is 2158 bp long and consists of six exons. *HOTAIR* orthologs are restricted to eutherian mammals [7]. *HOTAIR* is known to bind to the Polycomb Repressive Complex 2 (PRC2) and the histone H3K4 demethylase LSD1, and serves as a scaffold to assemble these regulators at the *HOXD* gene cluster, where it establishes a transcriptionally repressive chromatin structure, thereby resulting in epigenetic repression of the *HOXD* gene locus [8]. *HOTAIR* has been shown to function as an oncogene since its expression is dysregulated in multiple types of cancers, including breast, lung, liver, renal, hepatocellular, gastric, nasopharyngeal, cervical, colorectal, bladder, pancreatic cancer, as well as melanoma, leukemia, etc. [9,10,11,12,13]. Furthermore, *HOTAIR* is suggested to promote cancer progression and contribute largely to cancer cell invasion and metastasis [14,15,16,17]. The multifunctional *HOTAIR* is implicated in the different aspects of cancer pathophysiology by regulating gene expression at the transcriptional, post-transcriptional, and epigenetic level [14,18,19,20]. Of note, several studies suggest that *HOTAIR* expression is highly predictive of cancer patient survival rates in diverse cancer types [21,22,23,24,25,26,27,28,29].

Herein, we conducted a comprehensive and updated meta-analysis to further investigate the prognostic value of *HOTAIR* expression for cancer patients. The potential clinical applications of our findings are also discussed towards the prognostic application of *HOTAIR* to multiple and different types of cancers.

## 2. Results

### 2.1. Study Selection and Charasteristics of Eligible Studies

**Figure 1:**
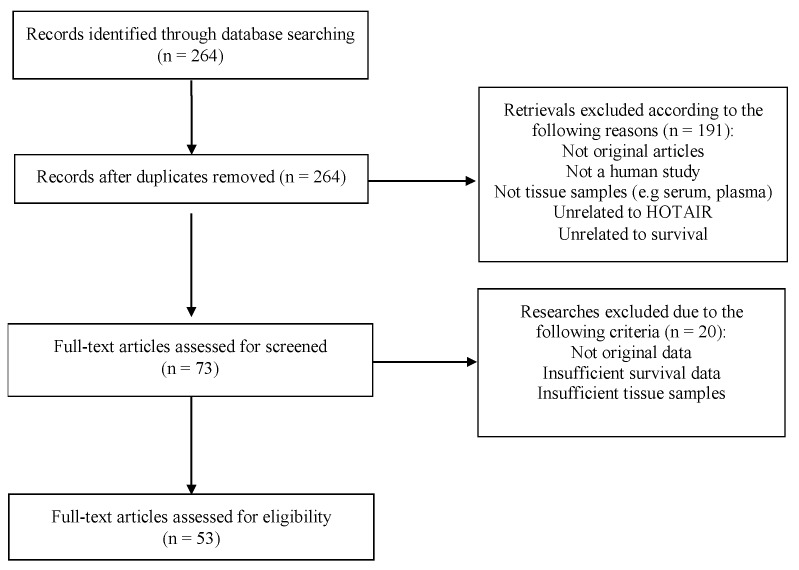
Flow chart of the process for study selection.

A total of 264 relevant published scientific studies were retrieved from the biomedical literature (up to 31 December 2018). According to the inclusion and exclusion criteria, 53 studies were ultimately included in this meta-analysis, as shown in Figure 1. The main characteristics of the included studies are summarized in Table 1, where the following information was recorded: first author’s surname; year of publication; country of origin; type of cancer; follow-up period (in months); total number of patients; detection assay for *HOTAIR* expression; HR and the corresponding 95% CI for overall survival (OS), recurrence-free survival (RFS), disease-free survival (DFS), progression-free survival (PFS), metastasis-free survival (MFS); survival data extraction method; and specimen type. Collectively, 4873 patients from 55 cohorts between 2010 and 2018 were included. The included studies reported a follow-up period ranging from 36 to 276 months. The level of *HOTAIR* expression was measured with quantitative reverse transcription polymerase chain reaction (qRT-PCR) in all of the included studies, except one where *HOTAIR* expression was estimated by microarrays (Table 1).

### 2.2. Association between High HOTAIR Expression and Overall Survival in Diverse Cancers

**Figure 2:**
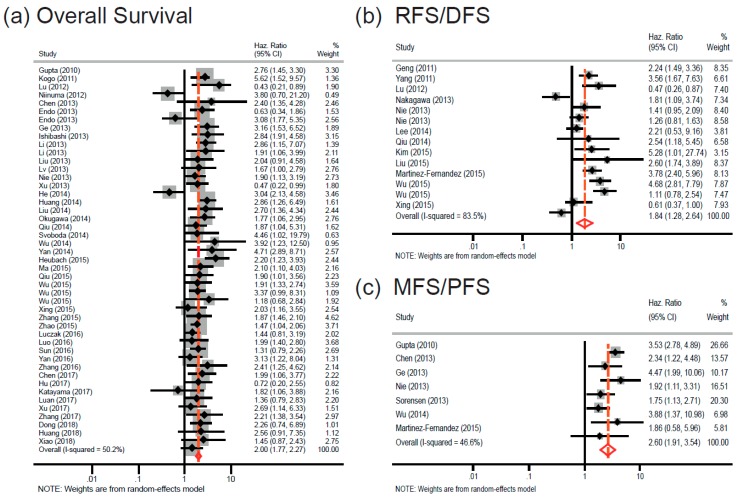
Forest plots of combined analyses on the association of survival with *HOTAIR* expression. (**a**) Forest plot of OS analysis, (**b**) forest plot of RFS/DFS analysis, and (**c**) forest plot of MFS/PFS analysis. Abbreviations: HR, Hazard ratio; OS, overall survival; RFS, recurrence-free survival; DFS, disease-free survival; MFS, metastasis-free survival; and PFS, progression-free survival.

**Figure 3:**
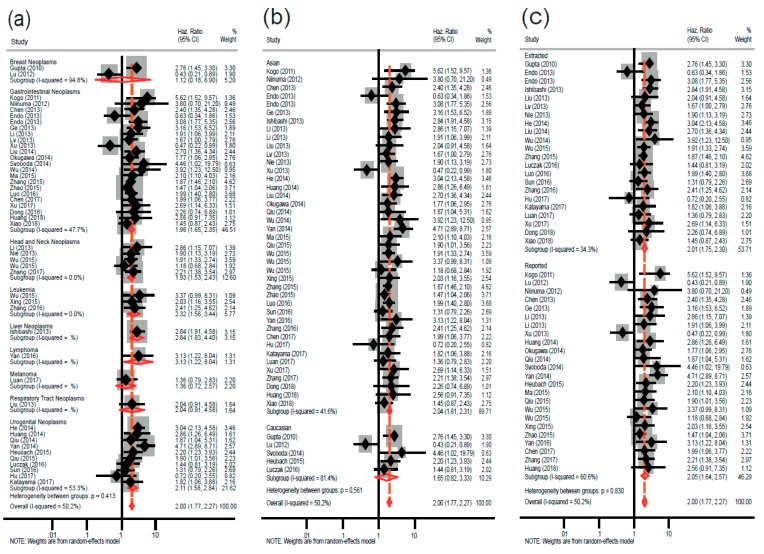
Forest plots of combined analyses for overall survival (OS) associated with *HOTAIR* expression in different groups. (**a**) Forest plot for different types of cancers, (**b**) forest plot for different ethnic groups, and (**c**) forest plot for different data extraction methods.

A total of 45 studies were included for overall survival (OS). We found a statistically significant relationship between elevated *HOTAIR* expression and poor OS (random-effects model: pooled HR = 2.00; 95% CI: 1.77–2.27; *p* < 0.001), with marginally moderate heterogeneity (I^2^ = 50.2%; P_h_ < 0.001) (Figure 2a). Subgroup analyses were performed based on the type of cancers, ethnic group, and data extraction method (Figure 3). When the studies were classified based on major cancer types (according to NCBI’s medical subject headings (MeSH) [77]), a significant association was found between *HOTAIR* overexpression and poorer OS in solid cancers, such as gastrointestinal cancers (fixed-effects model: pooled HR = 1.96; 95% CI: 1.65–2.35; *p* < 0.001), liver cancers (fixed-effects model: pooled HR = 2.84; 95% CI: 1.83–4.40; *p* < 0.001), head and neck cancers (fixed-effects model: pooled HR = 1.93; 95% CI: 1.53–2.43; *p* < 0.001), and urogenital cancers (random-effects model: pooled HR = 2.11; 95% CI: 1.58–2.84; *p* < 0.001), as well as liquid cancers, including leukemia (fixed-effects model: pooled HR = 2.32; 95% CI: 1.56–3.44; *p* < 0.001) and lymphoma (fixed-effects model: pooled HR = 3.13; 95% CI: 1.22–8.04; *p* < 0.001). Of note, the heterogeneity was reduced significantly in the individual cancer types (Figure 3a). In the subgroup analysis based on ethnicity, a statistically significant worse OS was observed for Asians (fixed-effects model: pooled HR = 2.04; 95% CI: 1.81–2.31; *p* < 0.001). Regarding the Caucasian subgroup, despite the relatively high HR, the relationship cannot be considered robust because the *p*-value is slightly higher that the cutoff value (random-effects model; pooled HR = 1.65; 95% CI: 0.82–3.33; *p* = 0.077) (Figure 3b). In stratified analysis, according to data extraction method, *HOTAIR* was found to have a significant prognostic value irrespectively of the data source. that is, the HR reported in the articles (random-effects model: pooled HR = 2.05; 95% CI: 1.64–2.57; *p* < 0.001) or extracted from the survival curves (fixed-effects model: pooled HR = 2.01; 95% CI: 1.75–2.30; *p* < 0.001) (Figure 3c).

### 2.3. HOTAIR Overexpression Is Associated with Cancer Recurrence and Progression

To investigate the relationship between *HOTAIR* expression and cancer recurrence or relapse, the recurrence-free survival (RFS) and disease-free survival (DFS) studies were combined; collectively accounting for 14 studies. Increased *HOTAIR* expression was found to be strongly related to cancer recurrence (pooled HR = 1.84; 95% CI = 1.28–2.64; *p* = 0.001). A random-effects model was applied because of the high heterogeneity (I^2^ = 83.5%; P_h_ < 0.001) across studies (Figure 2b). 

Furthermore, there are seven studies for combined metastasis-free survival (MFS) and progression-free survival (PFS). Of importance, high *HOTAIR* expression was predicted to be associated significantly with worse MFS/PFS (pooled HR = 2.60; 95% CI: 1.91–3.54; *p* < 0.001). A fixed-effects model was used because of the relatively low heterogeneity (I^2^ = 46.6%; P_h_ = 0.081) (Figure 2c).

### 2.4. Publication Bias

**Figure 4:**
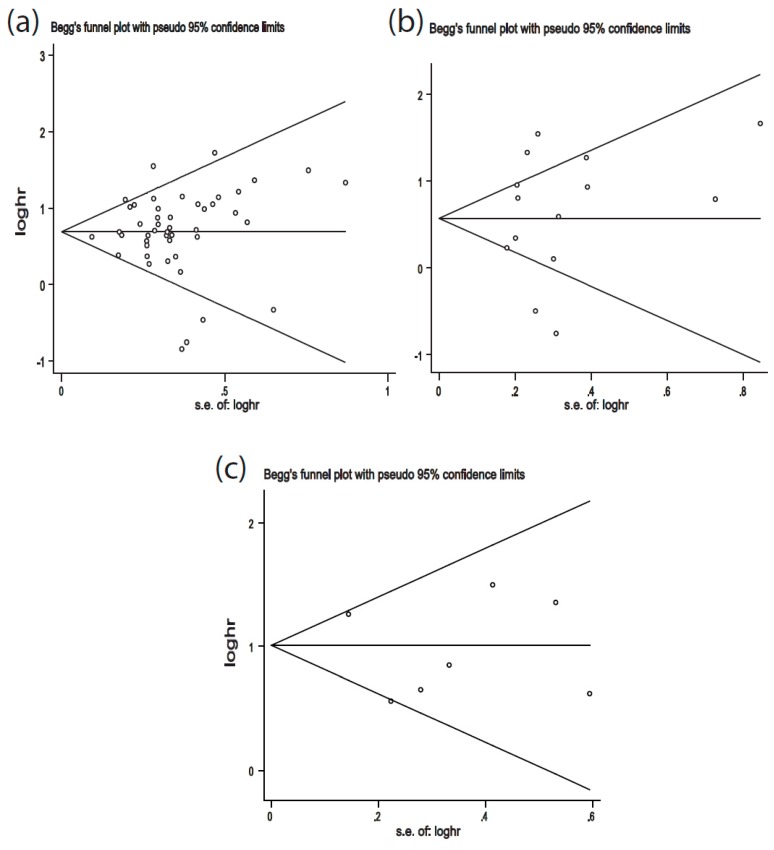
Begg’s funnel plots of publication bias. (**a**) Begg’s funnel plot of publication bias for OS; (**b**) Begg’s funnel plot of publication bias for RFS/DFS; (**c**) Begg’s funnel plot of publication bias for MFS/PFS. Each circle represents a separate study.

Publication bias was detected by Begg’s funnel plot and Egger’s test. There was no obvious asymmetry in Begg’s funnel plots of OS, RFS/DFS, and MFS/PFS (Figure 4). Additionally, the *p*-values of Egger’s tests were all greater than 0.05, indicating no potential publication bias (OS: *p* = 0.73; RFS/DFS: *p* = 0.70; MFS/PFS: *p* = 0.64).

### 2.5. Sensitivity Analysis

**Figure 5:**
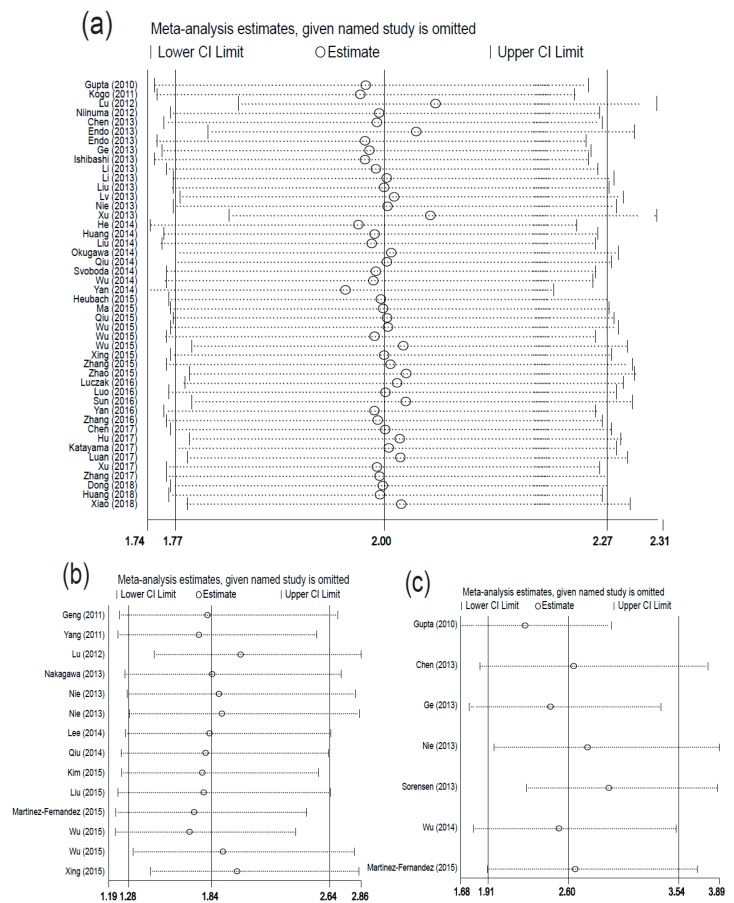
Sensitivity analysis of each eligible study. (**a**) OS individual studies, (**b**) RFS/DFS individual studies and (**c**) MFS/PFS individual studies.

Sensitivity analyses did not indicate alterations in the results due to the inclusion of any individual study (Figure 5), that is, no single study affected the pooled HR or 95% CI. 

### 2.6. TCGA-Derived Survival Curves

To further the clinical relevance of our work and *HOTAIR* importance, we explored the possibility for any association of the *HOTAIR* expression to overall cancer survival. It was found that *HOTAIR* overexpression was significantly associated with worse OS in adrenocortical carcinoma (ACC), mesothelioma (MESO), and glioblastoma multiforme (GBM) (Figure S1). 

## 3. Discussion

*HOTAIR* exhibits pro-oncogenic activity since it has been shown to be overexpressed in numerous cancers and be implicated in several hallmarks of cancer, such as cellular proliferation, inhibition of apoptosis, genomic instability, angiogenesis, invasion, and metastasis [19,20].

In the current study, an updated, comprehensive meta-analysis on the prognostic value of *HOTAIR* in various human cancers was presented. By applying stringent inclusion and exclusion criteria, we included 53 eligible studies, a relatively large number necessary for a meta-analysis to be considered robust. Previous meta-analyses on the association of *HOTAIR* with clinical outcome have included a rather limited number of studies with inconclusive and inconsistent findings [28,29]. Other related studies have focused on certain types of cancers, such as head and neck squamous cell carcinoma [22] or digestive system cancers [55,78,79].

In the present study, we showed that there is a statistically significant relationship between elevated *HOTAIR* expression and poor OS. In the subgroup analysis, based on cancer type, *HOTAIR* was shown to be a significant predictor for worse prognosis for a variety of cancers, including solid cancers, such as urological cancers, head and neck neoplasms, cancers of the digestive system, and several female cancers (e.g., cervical, ovarian, and endometrial cancers), as well as the blood cancers, lymphoma and leukemia. Moreover, we complemented the findings from meta-analysis and further strengthened our hypotheses with survival information from other types of cancers, for which there were not any available eligible studies, retrieved from TCGA. It was found that there is, also, a strong relationship between *HOTAIR* overexpression and poor OS in neoplasms of the adrenal cortex, mesothelial neoplasms, and neuroepithelial tumors.

Taken together, the above findings lead to the suggestion that similar *HOTAIR*-mediated pathways might be implicated both in solid and liquid cancers [13]. In particular, in several solid tumors, *HOTAIR* has been shown to exert its oncogenic and metastatic potential by mediating a repressive chromatin structure through the recruitment of histone-modifying or chromatin-remodeling complexes, such as PRC2 [14,16,31]. For example, *HOTAIR* can promote pancreatic cancer cell proliferation by suppressing the expression of miR-663b via remodeling the chromatin structure within the miR-663b promoter [80]. In a recent study, *HOTAIR* was also found to recruit PRC2 to catalyze H3K27 trimethylation to transcriptionally repress *E-cadherin* and promote EMT in gastric cancer [81]. Similarly, high expression levels of *HOTAIR* and PRC2 proteins (H3K27 methylase EZH2, SUZ12, and EED) were found to be positively correlated with lymphomagenesis [82]. In addition, *HOTAIR*, through miRNA sponging, contributes to carcinogenesis both in blood [60] and solid tumors [83,84]. However, there is a rather limited number of studies available on major cancers, such as breast neoplasms and respiratory tract cancers. Thus, more clinical trials on these cancers would enable us to better assess the relationship between *HOTAIR* expression and cancer patients’ survival.

A positive correlation between *HOTAIR* and *CDKN1A* (*p21*) expression levels was also found (Figure S2), suggesting a possible functional and/or physical association between *HOTAIR* and *CDKN1A* (*p21*) in cancer pathophysiology. From a clinical perspective, there is an emerging role of *CDKNIA* (*p21*), especially in cases where p53 is mutated like in many different solid tumors. The role of *p21* has been extensively viewed as an indicator of wildtype p53 activity [85]. However, recent evidence suggests that upregulated *p21* can also act as an oncogenic factor in a p53-deficient environment, thereby driving a subset of atypical cancerous cells to more chemoresistant and aggressive phenotypes [86]. Therefore, we cannot exclude a possible mechanistic association between *HOTAIR* and *p21* towards the negative regulation of target genes and a potential role in OS. Interestingly, recent studies have shown that *HOTAIR* expression was significantly higher in non-small-cell lung cancer (NSCLC) tissues compared to the adjacent normal tissues, and *HOTAIR* was negatively associated with p53 functionality rather than *p53* expression [87]. In addition, *HOTAIR*, *p21*, and *p53* mRNA expression in doxorubicin- or γ rays-treated oral squamous cell carcinoma (OSCC) cells was up-regulated, indicating that the DNA damage response includes *HOTAIR* upregulation and may be closely connected to *p53* and *p21* expression and/or functionality [88]. 

To investigate any possible effect of the genetic background and environment on the overall HRs, analyses were conducted based on the ethnic background of the participants. *HOTAIR* was found to be a powerful negative prediction biomarker for Asians. In the case of Caucasians, there was a link between *HOTAIR* overexpression and poor OS, albeit with moderate statistical significance; this is probably due to the relatively low number of available studies on patients of Caucasian origin. There were not, also, any available studies for other major ethnic groups, such as Africans or Indians, which would have further allowed us to estimate the influence of the genetic make-up on the association between *HOTAIR* and clinical outcome. The overall effect was similar in the stratified analysis according to data source, that is, the estimated HR reported in the articles or extrapolated from survival curves.

Therefore, high *HOTAIR* expression can predict an unfavorable clinical outcome in different types of cancers and possibly ethnic groups using different extraction methods. Notably, elevated expression of *HOTAIR* and prognosis in cancer patients is not particularly affected either by cancer type or even the patients’ genetic background.

*HOTAIR* was found to be a poor predictor for both cancer recurrence and progression. The similar outcomes suggest that there are similar *HOTAIR*-dependent mechanisms underlying these two phenomena. In particular, *HOTAIR* was shown to mediate recurrence and progression in bladder cancer via the histone methyltransferase EZH2 [56]. Similarly, enhanced *HOTAIR* expression was found to be associated both with progression and tumor recurrence in hepatocellular carcinoma by regulating the Wnt/β-catenin signal transduction pathway [89]. 

*HOTAIR* has been demonstrated to promote tumor cell invasion and metastasis by modulating epithelial-to-mesenchymal transition (EMT) [16,46,90]. Enhanced *HOTAIR* expression has also been shown to promote metastasis and invasion through different mechanisms including genome-wide re-targeting of PRC2 and subsequent epigenetic silencing of multiple anti-metastatic genes [14], inhibition of the expression of the metastasis suppressor gene *E-cadherin* by recruiting the histone methyltransferase of PRC2, EZH2 [16,90], targeting of Notch/Wnt signaling pathway-associated genes [91], and upregulating chondroitin sulfotransferase CHST15 [92], etc. *HOTAIR* also promotes invasion and migration by acting as a ‘miRNA sponge’, through targeting the corresponding miRNAs in the miR-1/CCND2 [93], miR-148a/SNAIL2 [72], and miR-23b/MAPK1 [94] axes.

Heterogeneity was observed within the forest plots of OS and RFS/DFS, suggesting that HRs vary across studies. For this reason, the random-effects model was applied, where the overall HR was estimated based on the weighted average of the HRs of the individual studies. Given that the overall effect for OS and RFS/DFS was not affected by any single study, according to sensitivity analyses, we could suggest that, despite heterogeneity, the pooled HR can be considered quite reliable and representative. 

Moreover, potential publication bias was not detected in the present meta-analysis, probably due to the sufficient representation of eligible studies in this meta-analysis.

## 4. Materials and Methods

### 4.1. Search Strategy and Study Eligibility Criteria

This systematic review and meta-analysis was conducted by following strictly the PRISMA (preferred reporting items for systematic reviews and meta-analyses) guidelines [95]. 

The bibliographic database PubMed/MEDLINE [96] was manually searched for published scientific studies on the associations between *HOTAIR* expression and prognosis in different types of cancers by using combinations of the relevant keywords: (“HOTAIR” OR “HOX transcript antisense RNA” or “HOXC cluster antisense RNA 4” or “HOXC-AS4” OR “HOXC11-AS1”) and (“cancer” or “carcinoma” or “tumor” or “neoplasm” or “malignancy”) and (“prognosis” or “survival” or “outcome” or “mortality” or “death”). The studies had to fulfill the following inclusion criteria so as to be considered eligible: (1) studies of human clinical trials, (2) studies including more than 30 patients in total, (3) the correlation between *HOTAIR* expression and cancer patients’ survival was estimated, (4) availability of HR and 95% confidence interval (CI) or survival curves or sufficient data to calculate HR and 95% CI, (5) quantitative measurement (e.g., qPCR) of *HOTAIR* expression in cancers was included, and (6) studies published in English. Accordingly, the studies were excluded on the basis of the following exclusion criteria: (1) laboratory studies on animal models or cell lines; (2) reviews, meta-analyses, editorials, case reports, commentaries, unpublished data; (3) lack of sufficient data to estimate HR and 95% CI; and (4) samples other than tissue (e.g., blood, serum).

### 4.2. Study Selection, Data Extraction, and Quality Assessment

All potential studies were independently retrieved from the literature by two of the authors (H.I.T. and D.O.). Quality assessment of the studies was performed by H.I.T. and D.O. independently. Any disagreement was resolved by a third investigator (A.P.). Relevant data were extracted from the included studies and recorded into an ad hoc Excel worksheet. In the case that the HR was not reported in the corresponding article, the data were extracted from the graphical survival plots (i.e., Kaplan-Meier curves) by using the Engauge Digitizer v10.11 software, as previously described [97]. 

### 4.3. Statistical Analyses

All statistical analyses were performed with STATA statistical software version 13.0 (Stata Corporation, College Station, TX, USA) and Microsoft Excel. The heterogeneity among the included studies was estimated by Higgins I-squared (I^2^) statistic as follows: I^2^ < 25%; no heterogeneity; 25% < I^2^ < 50%: low heterogeneity; 50% < I^2^ < 75%: moderate heterogeneity; I^2^ >75% high heterogeneity [98,99]. In the case of statistically significant heterogeneity (I^2^ > 50% and P_h_ < 0.05), a random-effect model was applied, otherwise a fixed-effect model [100,101] was used. Sensitivity analysis was performed by consecutive omission of individual studies to verify the consistency of outcomes. Potential publication bias was detected by Begg’s funnel plot [102] and Egger’s test [103]; a *p*-value less than 0.05 was indicative of statistically significant publication bias.

### 4.4. Bioinformatics Analysis

#### 4.4.1. Survival Analysis

Overall survival curves for different types of cancers were retrieved through the online tool GEPIA (Gene Expression Profiling Interactive Analysis) [104], which provides survival analysis based on datasets obtained from The Cancer Genome Atlas (TCGA) (https://tcga-data.nci.nih.gov).

#### 4.4.2. Correlation Analysis

Correlation analysis between gene expression levels was performed through the web-based tool GEPIA [104] which analyzes gene expression based on RNA sequencing (RNA-Seq) data from TCGA.

## 5. Conclusions

In this study, we have performed a meta-analysis complemented with bioinformatics analyses towards investigating the prognostic potential of the prominent lncRNA *HOTAIR* in cancer. On the basis of our findings, *HOTAIR* represents a potential powerful predictor of prognosis of overall survival, cancer recurrence, progression, and metastasis in multiple and diverse types of cancers. Therefore, *HOTAIR* could be applied in the clinical setting as a universal biomarker for monitoring cancer patient survival.

## Figures and Tables

**Table 1:** Main characteristics of the studies included in the meta-analysis.

Author, Year	Country	Cancer	Max. Follow-Up (Months)	Sample	Case Number			OS		DFS/RFS		MFS/PFS		Assay Method	Data Extraction Method
					High Expression	Low Expression	Total	HR (95% CI)	*p*-Value	HR (95%CI)	*p*-Value	HR (95% CI)	*p*-Value		
Gupta, 2010 [14]	USA	Breast Cancer	240	Tissue	44	88	132	2.76 (1.45–3.3)	0.036	NM	NM	3.53 (2.78–4.89)	0.017	qRT-PCR	K-M
Geng, 2011 [30]	China	HCC	36	Tissue	NM	NM	50	NM	NM	2.24 (1.49–3.36)	0,049	NM	NM	qRT-PCR	K-M
Kogo, 2011 [31]	Japan	CRC	60	Tissue	20	80	100	5.62 (1.52–9.57)	0.008	NM	NM	NM	NM	qRT-PCR	reported
Yang, 2011 [32]	China	HCC	45	Tissue	32	28	60	NM	NM	3.56 (1.67–7.63)	0.001	NM	NM	qRT-PCR	reported
Lu, 2012 [33]	Italy	Breast Cancer	108	Tissue	NM	NM	336	0.43 (0.21–0.89)	0.022	0.47 (0.26–0.87)	0.016	NM	NM	qRT-PCR	reported
Niinuma, 2012 [34]	Japan	GIST	200	Tissue	11	28	39	3.8 (0.7–21.2)	0.123	NM	NM	NM	NM	qRT-PCR	reported
Chen, 2013 [24]	China	ESCC	60	Tissue	27	51	78	2.40 (1.35–4.28)	0.003	NM	NM	2.34 (1.22–4.48)	0.01	qRT-PCR	reported
Endo, 2013 [17]	Japan	IGC	68	Tissue	23	13	36	0.63 (0.34–1.86)	0.137	NM	NM	NM	NM	qRT-PCR	K-M
Endo, 2013 [17]	Japan	DGC	60	Tissue	20	12	32	3.08 (1.77–5.35)	<0.01	NM	NM	NM	NM	qRT-PCR	K-M
Ge, 2013 [35]	China	ESCC	100	Tissue	90	47	137	3.16 (1.53–6.52)	0.002	NM	NM	4.47 (1.99–10.06)	0.001	qRT-PCR	reported
Ishibashi, 2013 [36]	Japan	HCC	36	Tissue	13	51	64	2.84 (1.91–4.58)	0.041	NM	NM	NM	NM	qRT-PCR	K-M
Li, 2013 [37]	China	LSCC	60	Tissue	33	39	72	2.86 (1.15–7.07)	0.023	NM	NM	NM	NM	qRT-PCR	reported
Li, 2013 [38]	China	ESCC	60	Tissue	30	70	100	1.91 (1.06–3.99)	0.033	NM	NM	NM	NM	qRT-PCR	reported
Liu, 2013 [39]	China	NSCLC	60	Tissue	21	21	42	2.043 (0.91–4.58)	0.048	NM	NM	NM	NM	qRT-PCR	K-M
Lv, 2013 [40]	China	ESCC	70	Tissue	49	44	93	1.67 (1.02–2.79)	0.049	NM	NM	NM	NM	qRT-PCR	K-M
Nakagawa, 2013 [21]	Japan	NSCLC	50	Tissue	17	60	77	NM	NM	1.81 (1.09–3.74)	0,047	NM	NM	qRT-PCR	K-M
Nie, 2013 [41]	China	NPC	82	Tissue	91	69	160	1.9 (1.13–3.19)	0.012	1.41 (0.95–2.09)	0.47	1.92 (1.11–3.31)	0.018	qRT-PCR	K-M
Sorensen, 2013 [42]	Denmark	Breast Cancer	276	Tissue	79	85	164	NM	NM	NM	NM	1.75 (1.13–2.71)	0.012	Microarray	reported
Xu, 2013 [43]	China	Gastric cancer	75	Tissue	56	27	83	0.47 (0.22–0.99)	0.04	NM	NM	NM	NM	qRT-PCR	reported
He, 2014 [44]	China	EC	48	Tissue	62	83	145	3.04 (2.13–4.58)	0.026	NM	NM	NM	NM	qRT-PCR	K-M
Huang, 2014 [45]	China	Cervical cancer	55	Tissue	109	109	218	2.86 (1.26–6.49)	0.012	NM	NM	NM	NM	qRT-PCR	reported
Lee, 2014 [46]	Korea	Gastric cancer	48	Tissue	28	20	48	NM	NM	2.21 (0.53–9.16)	0.141	NM	NM	qRT-PCR	reported
Liu, 2014 [18]	China	Gastric cancer	48	Tissue	39	39	78	2.7 (1.36–4.34)	0.023	NM	NM	NM	NM	qRT-PCR	K-M
Okugawa, 2014 [47]	Japan	Gastric cancer	60	Tissue	77	73	150	1.77 (1.06–2.95)	0.028	NM	NM	NM	NM	qRT-PCR	reported
Qiu, 2014 [48]	China	EOC	79	Tissue	32	32	64	1.87 (1.04–5.31)	0.041	2.54 (1.18–5.45)	0.034	NM	NM	qRT-PCR	reported
Svoboda, 2014 [49]	Czech Republic	Colorectal cancer	54	Tissue	36	37	73	4.46 (1.02–19.79)	0.048	NM	NM	NM	NM	qRT-PCR	reported
Wu, 2014 [50]	China	Colon Cancer	72	Tissue	40	80	120	3.92 (1.23–12.50)	0.021	NM	NM	3.88 (1.37–10.98)	0.011	qRT-PCR	K-M
Yan, 2014 [51]	China	Bladder Cancer	60	Tissue	90	20	110	4.71 (2.89–8.71)	<0.001	NM	NM	NM	NM	qRT-PCR	reported
Heubach, 2015 [52]	Germany	UHC	200	Tissue	27	81	108	2.20 (1.23–3.93)	0.008	NM	NM	NM	NM	qRT-PCR	reported
Kim, 2015 [53]	Korea	Cervical cancer	60	Tissue	89	22	111	NM	NM	5.28 (1.01–27.74)	0,049	NM	NM	qRT-PCR	reported
Liu, 2015 [54]	China	Gastric cancer	40	Tissue	24	37	61	NM	NM	2.6 (1.74–3.89)	<0.001	NM	NM	qRT-PCR	K-M
Ma, 2015 [55]	China	Gastric cancer	60	Tissue	18	53	71	2.10 (1.10–4.03)	0.022	NM	NM	NM	NM	qRT-PCR	reported
Martinez-Fernandez, 2015 [56]	Spain	NMIBC	38	Tissue	17	16	33	NM	NM	NM	NM	1.86 (0.58–5.96)	0.296	qRT-PCR	K-M
Martinez-Fernandez, 2015 [56]	Spain	NMIBC	38	Tissue	30	33	63	NM	NM	3.78 (2.40–5.96)	<0.001	NM	NM	qRT-PCR	K-M
Qiu, 2015 [57]	China	SOC	96	Tissue	34	34	64	1.90 (1.01–3.56)	0.046	NM	NM	NM	NM	qRT-PCR	reported
Wu, 2015 [58]	China	OSCC	60	Tissue	25	25	50	1.91 (1.33–2.74)	<0.001	NM	NM	NM	NM	qRT-PCR	K-M
Wu, 2015 [59]	China	AML	40	Tissue	52	33	85	3.37 (0.99–8.31)	0.008	4.68 (2.81–7.79)	<0.001	NM	NM	qRT-PCR	reported
Wu, 2015 [16]	China	OSCC	96	Tissue	38	38	76	1.18 (0.68–2.84)	0.03	1.11 (0.78–2.54)	0.044	NM	NM	qRT-PCR	reported
Xing, 2015 [60]	China	AML	36	Tissue	68	68	136	2.03 (1.16–3.55)	0.007	0.61 (0.37–1.00)	0.034	NM	NM	qRT-PCR	reported
Zhang, 2015 [61]	China	Gastric cancer	45	Tissue	35	15	50	1.87 (1.46–2.1)	0.028	NM	NM	NM	NM	qRT-PCR	K-M
Zhao, 2015 [62]	China	Gastric cancer	65	Tissue	84	84	168	1.47 (1.04–2.06)	0.027	NM	NM	NM	NM	qRT-PCR	reported
Luczak, 2016 [63]	Poland	EC	96	Tissue	56	100	156	1.44 (0.81–3.19)	0.03	NM	NM	NM	NM	qRT-PCR	K-M
Luo, 2016 [64]	China	Colon cancer	70	Tissue	NM	NM	80	1.99 (1.4–2.8)	<0.001	NM	NM	NM	NM	qRT-PCR	K-M
Sun, 2016 [65]	China	Cervical cancer	50	Tissue	49	10	59	1.31 (0.79–2.26)	0.02	NM	NM	NM	NM	qRT-PCR	K-M
Yan, 2016 [66]	China	DLBCL	120	Tissue	25	25	50	3.13 (1.22–8.04)	0.018	NM	NM	NM	NM	qRT-PCR	reported
Zhang, 2016 [67]	China	Acute leukemia	40	Tissue	19	77	96	2.41 (1.25–4.62)	0.005	NM	NM	NM	NM	qRT-PCR	K-M
Chen, 2017 [68]	China	Gastric cancer	62	Tissue	33	32	65	1.99 (1.06–3.77)	0.033	NM	NM	NM	NM	qRT-PCR	reported
Hu, 2017 [69]	China	RCC	50	Tissue	32	11	43	0.72 (0.20–2.55)	0.62	NM	NM	NM	NM	qRT-PCR	K-M
Katayama, 2017 [70]	Japan	RCC	100	Tissue	21	43	64	1.82 (1.06–3.88)	0.02	NM	NM	NM	NM	qRT-PCR	K-M
Luan, 2017 [71]	China	MM	60	Tissue	30	30	60	1.36 (0.79–2.83)	0.01	NM	NM	NM	NM	qRT-PCR	K-M
Xu, 2017 [72]	China	* EC	36	Tissue	20	20	40	2.69 (1.14–6.33)	0.032	NM	NM	NM	NM	qRT-PCR	K-M
Zhang, 2017 [73]	China	Thyroid cancer	60	Tissue	NM	NM	35	2.21 (1.38–3.54)	0.001	NM	NM	NM	NM	qRT-PCR	reported
Dong, 2018 [74]	China	Gastric cancer	60	Tissue	22	10	32	2.26 (0.74–6.89)	0.158	NM	NM	NM	NM	qRT-PCR	K-M
Huang, 2018 [75]	China	Colorectal cancer	110	Tissue	26	26	52	2.56 (0.91–7.35)	<0.01	NM	NM	NM	NM	qRT-PCR	reported
Xiao, 2018 [76]	China	Colorectal cancer	60	Tissue	52	52	104	1.45 (0.87–2.43)	0.041	NM	NM	NM	NM	qRT-PCR	K-M

Abbreviations: OS, overall survival; RFS, recurrence-free survival; DFS, disease-free survival; MFS, metastasis-free survival; PFS, progression-free survival; HR, hazard ratio; CI, confidence interval; qRT-PCR, quantitative reverse transcription polymerase chain reaction; NM: not mentioned; K-M, Kaplan-Meier plot; AML, acute myeloid leukemia; CRC, colorectal cancer; DGC, diffuse gastric cancer; DLBCL, diffuse large B cell lymphoma; ESCC, esophageal squamous cell carcinoma; EC, endometrial carcinoma; EOC, epithelial ovarian cancer; * EC, esophageal cancer; GIST, gastrointestinal stromal tumors; HCC, hepatocellular carcinoma; IGC, intestinal gastric cancer; LSCC, laryngeal squamous cell carcinoma; MM, malignant melanoma; NSCLC, non-small cell lung cancer; NPC, nasopharyngeal carcinoma; NMIBC, non-muscle-invasive bladder cancer; OSCC, oral squamous cell carcinoma; RCC, renal cell carcinoma; SOC, serous ovarian cancer; and UHC, urothelial carcinoma.

## Supplementary Materials

The following are available online at https://www.mdpi.com/2072-6694/11/6/778/s1, Figure S1: Kaplan-Meier plots depicting the prognostic potential of *HOTAIR* for OS in various types of cancers. (A) ACC; (B) MESO and (C) GBM. The corresponding HRs and *p*-values are indicated. The CIs are denoted by dashed lines, Figure S2: Correlation between *HOTAIR* and *CDKN1A* expression.
