# Supplementary material for: HOTAIR as a Prognostic Predictor for Diverse Human Cancers: A Meta- and Bioinformatics Analysis
Source: Cancers (Basel). 2019 Jun 5;11(6):778. doi: 10.3390/cancers11060778 (PMC6628152; doi:10.3390/cancers11060778)
Supplement: Supplementary file 1 [file cancers-11-00778-s001.pdf]

HOTAIR as a Prognostic Predictor for Diverse Human Cancers: A Meta- and Bioinformatics Analysis

Halil Ibrahim Toy, Didem Okmen, Panagiota I. Kontou, Alexandros G. Georgakilas and Athanasia Pavlopoulou

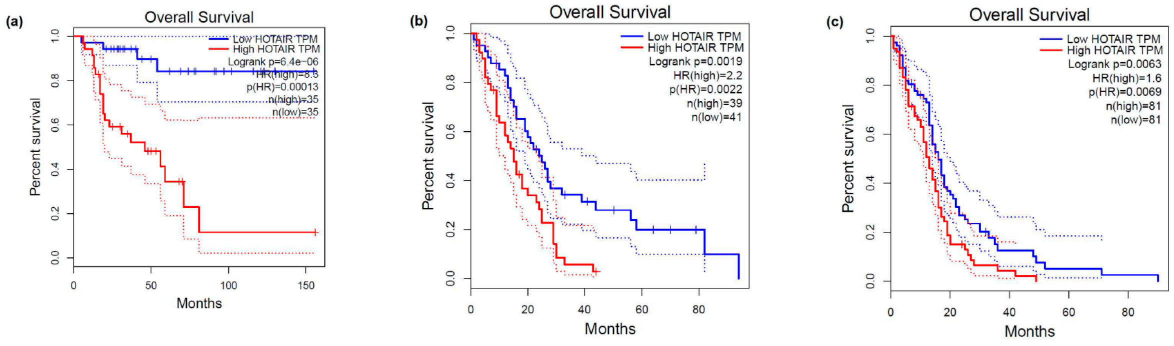

Figure S1. Kaplan–Meier plots depicting the prognostic potential of *HOTAIR* for OS in various types of cancers. (**a**) ACC; (**b**) MESO and (**c**) GBM. The corresponding HRs and *p*-values are indicated. The CIs are denoted by dashed lines.

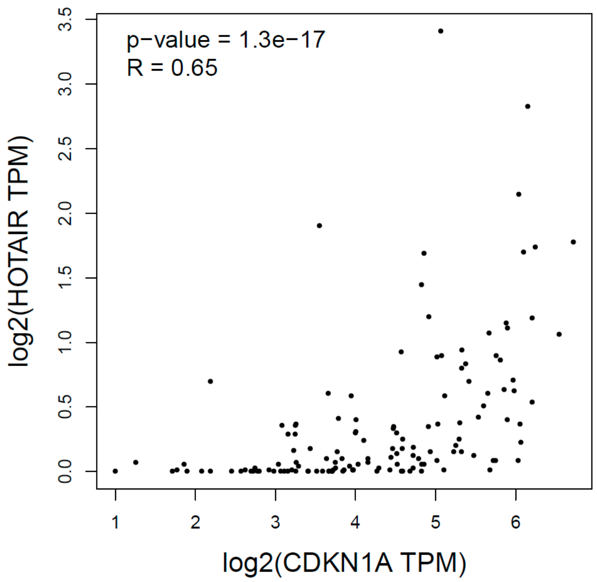

Figure S2. Correlation between *HOTAIR* and *CDKN1A* expression.

© 2019 by the authors. Licensee MDPI, Basel, Switzerland. This article is an open access article distributed under the terms and conditions of the Creative Commons Attribution (CC BY) license (http://creativecommons.org/licenses/by/4.0/).
